# Venous Thromboembolism and Risk of Cancer in Users of Low-Dose Aspirin: A Danish Population-Based Cohort Study

**DOI:** 10.1055/s-0042-1755606

**Published:** 2022-09-12

**Authors:** Gencer Kurt, Dávid Nagy, Frederikke S. Troelsen, Nils Skajaa, Rune Erichsen, Dóra K. Farkas, Henrik T. Sørensen

**Affiliations:** 1 Department of Clinical Epidemiology, Aarhus University Hospital and Aarhus University, Aarhus N, Denmark

**Keywords:** venous thromboembolism, low-dose aspirin, cancer, cohort

## Abstract

**Background** Aspirin may reduce the risk of cancer, particularly gastrointestinal cancer, and venous thromboembolism (VTE). VTE can be the first symptom of occult cancer, but whether it is also a marker of occult cancer in aspirin users remains unknown. Therefore, we investigated the risk of cancer subsequent to VTE among users of low-dose aspirin.

**Methods** We conducted a population-based cohort study using data from Danish health registries for the years 2001 to 2018. We identified all patients with a first-time diagnosis of VTE who also redeemed a prescription for low-dose aspirin (75–150mg) within 90 days prior to the first-time VTE. We categorized aspirin users by the number of prescriptions filled as new users (<5 prescriptions), short-term users (5–19 prescriptions), and long-term users (>19 prescriptions). We computed the absolute cancer risks and standardized incidence ratios (SIRs) for cancer using national cancer incidence rates.

**Results** We followed-up 11,759 users of low-dose aspirin with VTE. Long-term users comprised 50% of aspirin users. The 1-year absolute risk of cancer was 6.0% for new users and 6.7% for short-term and long-term users, with corresponding SIRs of 3.3 (95% confidence interval [CI]: 2.8–4.0), 3.2 (95% CI: 2.9–3.7), and 2.8 (95% CI: 2.6–3.2), respectively. After the first year of follow-up, the SIR decreased to 1.2 (95% CI: 1.1–1.4) for new users, 1.1 (95% CI: 1.1–1.3) for short-term users, and 1.1 (95% CI: 1.0–1.2) for long-term users.

**Conclusion** VTE may be a harbinger of cancer, even in users of low-dose aspirin, regardless of duration of use.

## Introduction

Venous thromboembolism (VTE), including deep vein thrombosis (DVT) and pulmonary embolism (PE), is a well-established complication of cancer 1 and associated with 1-year survival as low as 12%. 2 Conversely, VTE may also be the first symptom of occult cancer. 3 4 5 Several studies have reported that patients with VTE have up to a 5.2% risk of cancer within the first year after a VTE diagnosis which corresponds to a two- to four-fold increased risk of cancer compared with the general population. 3 4 5 6 7 8 The risk of VTE recurrence and VTE in selected surgical and medical patients may be reduced by aspirin use. 9

Long-term continuous use of aspirin may reduce the risk of some cancers, particularly gastrointestinal cancers, including colorectal, gastric, and esophageal cancer. 10 11 12 Plausible mechanisms contributing to the cancer-protective effects of aspirin include induction of cellular apoptosis and inhibition of cyclooxygenase-catalyzed prostaglandins which can promote tumor growth. 13

Low-dose aspirin is recommended as a preventative in patients who have survived an arterial cardiovascular event, such as myocardial infarction or stroke. 14 This is a group of patients suggested to be at an increased risk of VTE, 15 probably due to hypercoagulability, immobilization, and shared risk factors. 16 Furthermore, patients with an arterial cardiovascular event and subsequent VTE also have an increased risk of a cancer diagnosis. 17

Given that aspirin is associated with a reduced risk of both cancer and VTE, it is important to understand whether VTE is a marker of occult cancer in aspirin users and the extent to which the duration of aspirin use may affect the risk of cancer after the diagnosis of VTE. This knowledge may have potential clinical implications for the diagnostic workup of cancer among users of aspirin with VTE.

In this nationwide cohort study in Denmark, we investigated the risk of cancer subsequent to VTE diagnosis among users of low-dose aspirin by comparing the observed cancer incidence among aspirin users to the expected cancer incidence based on national cancer statistics. To evaluate the potential clinical implications of our results, we investigated the number of VTE patients needed to examine to detect one excess case of cancer within the first year after a VTE diagnosis.

## Methods

### Setting

We obtained data from the Danish Civil Registration System, 18 the Danish National Patient Registry (DNPR) covering all Danish hospitals, 19 the Danish National Prescription Registry (NPR), 20 and the Danish Cancer Registry (DCR) 21 to conduct this population-based cohort study within the entire Danish population covering January 1, 2001, through December 31, 2018. The cumulative source population size during the study period was approximately 7.3 million individuals. The Supplementary Tables S1 – S4 (available in the online version) provide a detailed description of data sources, with codes for VTE, drug exposure, cancer, and covariates.

In Denmark, all residents have universal access to tax-funded health care, 22 and the Danish Civil Registration System assigns a unique civil registration number to each resident at birth or upon immigration. 18 The civil registration number allows accurate and individual-level linkage of data between the Danish health registries.

This study was registered with the Danish Data Protection Agency on behalf of Aarhus University (record number: 2016–051–000001/811).

### Venous Thromboembolism

We searched the DNPR to identify all patients with a first-time inpatient or outpatient clinic diagnosis of VTE, including DVT and PE. We used both primary (main reason for hospitalization) and secondary (diagnoses supplementing the primary diagnosis) diagnosis codes. Patients with a diagnosis of any cancer recorded prior to the date of first-time VTE were excluded from the study.

### Low-Dose Aspirin Use

From the NPR, we identified individuals who redeemed prescriptions for low-dose aspirin (75, 100, or 150mg) within 90 days prior to the date of first-time VTE. We chose January 1, 2001, as the starting of the study period to ensure at least 6 years of prescription data before study entry (prescription data available since 1995). The Danish health care system provides partial reimbursement to all Danish residents for most prescribed medications, including low-dose aspirin. Aspirin is available over-the-counter, but approximately 90% of low-dose aspirin sales in Denmark are dispensed by prescription due to the reimbursement. 23

The effect of aspirin use on cancer risk is most pronounced after at least 5 years of use. 10 As low-dose aspirin is mainly prescribed in packages of 100 tablets, 24 we assumed that the number of prescriptions represented the number of days that low-dose aspirin was taken (i.e., one prescription is equal to 100 days of low-dose aspirin use); thus, <5, 5 to 19, or >19 filled prescriptions are equal to approximately <1 year, 1 to 5 years, or more than 5 years of low-dose aspirin use, respectively. Therefore, we categorized all aspirin users into three groups according to duration of use, defined as number of prescriptions filled prior to the date of first-time VTE diagnosis: new users (<5 prescriptions), short-term users (5–19 prescriptions), and long-term users (>19 prescriptions).

### Cancer Outcomes

The primary outcome was any diagnosis of cancer. Data on incident cancer recorded after the date of VTE diagnosis were obtained from the DCR. We categorized cancers according to the yearly cancer report from the Danish Health Data Authority. 25

As patients diagnosed with cancer within 1 year after a VTE event are more likely to have advanced disease, 2 we classified the cancer stage as localized, nonlocalized, or unknown, with the latter stage including non-solid tumors.

### Covariates

From the DNPR, we obtained data on the presence of provoking factors for VTE. Selected provoking factors were pregnancy, trauma/fractures, and surgery as recorded in the DNPR within 3 months prior to the VTE diagnosis. 1 To measure the burden of comorbidity, we searched the DNPR to obtain information on diagnoses included in the Charlson comorbidity index (CCI) recorded before the date of admission for VTE. 26 27 The CCI includes 19 diseases, and each disease is assigned between 1 and 6 points depending on the strength of the association with mortality. Using the calculated CCI score, we categorized the members of the study cohort into three subgroups: low (no comorbidities), CCI score of 0; medium, CCI score of 1 and 2; or high, CCI score ≥3. Notably, we applied a modified CCI excluding any tumors prior to the VTE diagnosis. In addition, assuming that low-dose aspirin is prescribed for primary prevention in high-risk patients with diabetes, 28 as well as secondary prevention in patients who have survived an arterial cardiovascular event, we grouped aspirin users according to ischemic stroke, myocardial infarction, and diabetes (types 1 and 2) diagnosed before the date of VTE diagnosis.

### Statistical Analysis

Aspirin users with VTE were characterized by sex, age at VTE diagnosis, and calendar period of VTE diagnosis. Age at VTE diagnosis was divided into two groups (0–60 years and ≥61 years), as the majority of aspirin users with VTE were>60 years old. The calendar period for VTE diagnosis was divided into three groups (2001–2006, 2007–2012, and 2013–2017) because the diagnostic accuracy of VTE and cancer, as well as awareness of the association between cancer and VTE, may have changed during the study period. 29

We followed-up aspirin users with VTE from the date of VTE until the occurrence of a first-time cancer diagnosis, death, emigration, or the end of the study (December 31, 2018), whichever came first. As the risk of cancer following a VTE diagnosis decreases after 1 year, 3 the follow-up period was divided into ≤1 year and >1 year following the VTE diagnosis.

The absolute risks of cancer were calculated by the Aalen–Johansen estimator of the cumulative incidence function, treating death as a competing risk. 30 31 The absolute risks of sex-specific cancers were calculated by restricting the study cohort to the relevant sex. Standardized incidence ratios (SIRs) describing the ratio of the observed to the expected number of cancers were used as a measure of the relative risk of cancer in aspirin users with VTE. The expected number of cancer cases was estimated using national incidence rates for first-time cancer diagnoses according to sex, age, and year of diagnosis (in 5-year intervals). We used Byar's approximation to calculate 95% confidence intervals (CIs), assuming that the observed number of cancers in a given category followed a Poisson's distribution. 32 When the observed number was <10, we applied the exact 95% CI. We stratified the analyses by type of VTE (DVT or PE). Absolute risks and SIRs were further stratified by sex, age, calendar period, comorbidity burden at the date of VTE, presence of select provoking factors for VTE, and cancer stage.

Under the assumption that cancers detected during the first year of follow-up were present at the time of VTE diagnosis, we calculated the number of VTE patients needed to examine to detect one excess cancer case as the reciprocal of the excess risk (i.e., the difference between the observed number of cancers and expected number of cancers divided by the follow-up time) and the corresponding 95% CIs as the reciprocal of the CI of the excess risk estimate. 33

To investigate whether the time from aspirin prescription to a VTE diagnosis affected our results, we repeated the analyses with VTE patients who redeemed prescriptions for low-dose aspirin within 120 days rather than 90 days prior to the date of first-time VTE. The results were consistent with the primary analyses and, therefore, not reported (data not shown).

## Results

### Descriptive Data

We followed-up 11,759 users of low-dose aspirin with VTE for a median of 3 years (interquartile range: 0.9–6.4 years). Among new, short-term, and long-term users, 49, 52, and 55%, respectively, were female. New users were, as expected, younger than short-term and long-term users at VTE diagnosis (median age: 69 years vs. 74 and 78 years), had a lower comorbidity burden (61 vs. 73 and 80% with medium-to-high comorbidity burden), and more likely to have one or more of the selected provoking factors for VTE (29 vs. 23 and 23%; Table 1 ).

**Table 1:** Characteristics of users of low-dose aspirin with venous thromboembolism

Characteristics	New users	Short-term users	Long-term users
	*n* (%)	*n* (%)	*n* (%)
VTE all	2,003 (100)	3,851 (100)	5,905 (100)
Deep vein thrombosis	1,092 (54.5)	2,091 (54.3)	2,862 (48.5)
Pulmonary embolism	911 (45.5)	1,760 (45.7)	3,043 (51.5)
Median follow-up time (IQR) in years	4.7 (1.4–8.8)	3.5 (0.9–7.2)	2.5 (0.7–5.3)
Sex			
Female	977 (48.8)	1,998 (51.9)	3,249 (55.0)
Male	1,026 (51.2)	1,853 (48.1)	2,656 (45.0)
Median age at VTE diagnosis (IQR) in years	69 (59–79)	74 (66–82)	78 (71–85)
Age at VTE diagnosis			
0–60 years	521 (26.0)	508 (13.2)	332 (5.6)
≥61 years	1,482 (74.0)	3,343 (86.8)	5,573 (94.4)
Year of VTE diagnosis			
2001–2006	849 (42.4)	1,472 (38.2)	1,168 (19.8)
2007–2012	739 (36.9)	1,522 (39.5)	2,403 (40.7)
2013–2017	415 (20.7)	857 (22.3)	2,334 (39.5)
Comorbidity burden a			
Low	776 (38.7)	1,031 (26.8)	1,197 (20.3)
Medium	926 (46.2)	2,010 (52.2)	2,949 (49.9)
High	301 (15.0)	810 (21.0)	1,759 (29.8)
Selected comorbidities			
Ischemic stroke	218 (10.9)	644 (16.7)	1,107 (18.8)
Myocardial infarction	305 (15.2)	735 (19.1)	1,607 (27.2)
Diabetes mellitus (types 1 and 2)	280 (14.0)	732 (19.0)	1,470 (24.9)
Provoking factor present b			
No	1,419 (70.8)	2,958 (76.8)	4,561 (77.2)
Yes	584 (29.2)	893 (23.2)	1,344 (22.8)

Abbreviations: IQR, interquartile range; VTE, venous thromboembolism.

a Based on Charlson's comorbidity index scores (low: 0, medium: 1–2, and high ≥3).

b Selected provoking factors were pregnancy, trauma/fractures, and surgery.

Among aspirin users with VTE, 2,003 (17%) were new users, 3,851 (33%) short-term users, and 5,905 (50%) long-term users. Among these individuals, 55, 54, and 48%, respectively, had DVT.

The distribution of selected comorbidities (ischemic stroke, myocardial infarction, and diabetes) was similar to the distribution of high comorbidity burden in the three cohorts of aspirin users ( Table 1 ). The number of aspirin users with and without a diagnosis of ischemic stroke, myocardial infarction, or diabetes was not equally distributed, so we did not include these diagnoses in further subgroup analyses.

### Incident Cancers

#### New Users

We observed 407 cancers among new users of low-dose aspirin, including 121 during the first year of follow-up, yielding an absolute risk of 6.0% ( Table 2 ). The absolute risk was even higher for patients >60 years of age at VTE diagnosis, patients with low comorbidity burden, and patients without the selected provoking factors.

**Table 2:** Absolute risk of cancer in users of low-dose aspirin during the first year subsequent to venous thromboembolism

	New users	Short-term users	Long-term users
Characteristics	AR % (95% CI)	AR % (95% CI)	AR % (95% CI)
All	6.0 (5.1–7.1)	6.7 (5.9–7.5)	6.7 (6.1–7.3)
Type of VTE			
Deep vein thrombosis	5.2 (4.0–6.7)	5.5 (4.6–6.5)	6.0 (5.2–6.9)
Pulmonary embolism	7.0 (5.5–8.8)	8.0 (6.8–9.3)	7.3 (6.4–8.3)
Sex			
Female	6.4 (4.9–8.0)	5.8 (4.8–6.8)	5.6 (4.9–6.5)
Male	5.8 (4.4–7.3)	7.6 (6.5–8.9)	8.0 (7.0–9.0)
Age at VTE diagnosis			
0–60 years	2.1 (1.1–3.6)	2.6 (1.4–4.2)	2.7 (1.3–4.9)
≥61 years	7.4 (6.2–8.8)	7.3 (6.4–8.2)	6.9 (6.3–7.6)
Year of VTE diagnosis			
2001–2006	5.9 (4.4–7.6)	6.0 (4.9–7.3)	5.4 (4.2–6.8)
2007–2012	6.2 (4.6–8.1)	7.4 (6.2–8.8)	6.9 (5.9–7.9)
2013–2017	6.0 (4.0–8.6)	6.4 (4.9–8.2)	7.1 (6.1–8.2)
Comorbidity burden a			
Low	7.9 (6.1–9.9)	9.0 (7.4–10.9)	8.8 (7.3–10.5)
Medium	5.2 (3.9–6.7)	6.0 (5.0–7.1)	6.8 (5.9–7.7)
High	4.0 (2.2–6.6)	5.2 (3.8–6.9)	5.1 (4.2–6.2)
Provoking factor present b			
No	7.0 (5.7–8.4)	6.5 (5.6–7.4)	7.1 (6.3–7.8)
Yes	3.8 (2.4–5.5)	7.3 (5.7–9.1)	5.4 (4.2–6.7)
Cancer stage			
Localized	1.3 (0.8–1.8)	1.5 (1.1–1.9)	1.8 (1.5–2.2)
Nonlocalized	2.2 (1.6–2.9)	2.9 (2.4–3.5)	2.8 (2.4–3.3)
Unknown	2.6 (2.0–3.4)	2.3 (1.9–2.8)	2.1 (1.7–2.5)

Abbreviations: AR, absolute risk; CI, confidence interval; VTE, venous thromboembolism.

a Based on Charlson's comorbidity index scores (low: 0, medium: 1–2, and high ≥ 3).

b Selected provoking factors were pregnancy, trauma/fractures, and surgery.

The SIR for new aspirin users was 3.3 (95% CI: 2.8–4.0) during the first year of follow-up and was generally highest among females, patients with low comorbidity burden, and patients without the selected provoking factors ( Table 3 ). The site-specific SIRs during the first year of follow-up were highest for cancers of the liver, pancreas, lung, and kidney, as well as the non-Hodgkin lymphoma ( Table 4 ). The overall SIR decreased to 1.2 (95% CI: 1.1–1.4) in the subsequent years of follow-up ( Table 5 ).

**Table 3:** Standardized incidence ratios for cancer in users of low-dose aspirin with venous thromboembolism during the first year of follow-up

	New users		Short-term users		Long-term users	
Characteristics	O/E	SIR (95% CI)	O/E	SIR (95% CI)	O/E	SIR (95% CI)
All	121/36	3.3 (2.8–4.0)	256/79	3.2 (2.9–3.7)	394/138	2.8 (2.6–3.2)
Type of VTE						
Deep vein thrombosis	57/20	2.8 (2.1–3.7)	115/46	2.5 (2.1–3.0)	172/71	2.4 (2.1–2.8)
Pulmonary embolism	64/16	4.0 (3.1–5.1)	141/33	4.2 (3.6–5.0)	222/67	3.3 (2.9–3.8)
Sex						
Female	62/16	3.9 (3.0–5.0)	115/36	3.2 (2.6–3.8)	183/66	2.8 (2.4–3.2)
Male	59/21	2.9 (2.2–3.7)	141/43	3.3 (2.8–3.9)	211/73	2.9 (2.5–3.3)
Age at VTE diagnosis						
0–60 years	11/4	3.2 (1.6–5.7)	13/4	3.4 (1.8–5.8)	9/3	3.0 (1.4–5.8)
≥61 years	110/33	3.4 (2.8–4.0)	243/75	3.2 (2.8–3.7)	385/135	2.8 (2.6–3.1)
Year of VTE diagnosis						
2001–2006	50/15	3.4 (2.5–4.5)	88/27	3.2 (2.6–4.0)	63/23	2.7 (2.1–3.5)
2007–2012	46/14	3.4 (2.5–4.5)	113/32	3.6 (2.9–4.3)	165/56	3.0 (2.5–3.5)
2013–2017	25/8	3.2 (2.1–4.8)	55/20	2.8 (2.1–3.6)	166/59	2.8 (2.4–3.3)
Comorbidity burden a						
Low	61/14	4.4 (3.4–5.6)	93/23	4.1 (3.3–5.1)	105/29	3.6 (3.0–4.4)
Medium	48/17	2.8 (2.1–3.7)	121/41	2.9 (2.4–3.5)	199/70	2.9 (2.5–3.3)
High	12/5	2.3 (1.2–4.0)	42/15	2.7 (2.0–3.7)	90/40	2.3 (1.8–2.8)
Provoking factor present b						
No	99/26	3.8 (3.1–4.7)	191/62	3.1 (2.7–3.6)	322/108	3.0 (2.7–3.3)
Yes	22/11	2.1 (1.3–3.2)	65/17	3.7 (2.9–4.8)	72/31	2.3 (1.8–3.0)
Cancer stage						
Localized	25/18	1.4 (0.9–2.1)	57/39	1.5 (1.1–1.9)	106/69	1.5 (1.3–1.9)
Nonlocalized	44/10	4.6 (3.3–6.2)	111/20	5.5 (4.5–6.6)	166/32	5.1 (4.4–6.0)
Unknown	52/9	5.9 (4.4–7.7)	88/20	4.4 (3.5–5.4)	122/37	3.3 (2.7–3.9)

Abbreviations: CI, confidence interval; O/E, observed/expected; SIR, standardized incidence ratio; VTE, venous thromboembolism.

a Based on Charlson's comorbidity index scores (low: 0, medium: 1–2, and high ≥ 3).

b Selected provoking factors were pregnancy, trauma/fractures, and surgery.

**Table 4:** Absolute cancer risk and site-specific standardized incidence ratios for cancer in users of low-dose aspirin with venous thromboembolism during the first year of follow-up

	New users a			Short-term users a			Long-term users a		
Cancer groups	O/E	AR % (95% CI)	SIR (95% CI)	O/E	AR % (95% CI)	SIR (95% CI)	O/E	AR % (95% CI)	SIR (95% CI)
All	121/36	6.0 (5.1–7.1)	3.3 (2.8–4.0)	256/79	6.7 (5.9–7.5)	3.2 (2.9–3.7)	394/138	6.7 (6.1–7.3)	2.8 (2.6–3.2)
Esophagus	–	–	–	–	–	–	5/1	0.1 (0.0–0.2)	3.5 (1.1–8.1)
Stomach	–	–	–	–	–	–	5/2	0.1 (0.0–0.2)	2.7 (0.9–6.3)
Colon, including colon rectosigmoid	13/3	0.7 (0.4–1.1)	4.8 (2.5–8.2)	25/6	0.7 (0.4–1.0)	4.0 (2.6–6.0)	39/11	0.7 (0.5–0.9)	3.5 (2.5–4.8)
Rectal	–	–	–	7/3	0.2 (0.1–0.4)	2.5 (1.0–5.1)	10/5	0.2 (0.1–0.3)	2.1 (1.0–3.8)
Liver	9/0	0.5 (0.2–0.8)	29.3 (13.4–55.7)	5/1	0.1 (0.1–0.3)	7.5 (2.4–17.5)	–	–	–
Pancreas	7/1	0.4 (0.2–0.7)	8.4 (3.4–17.2)	9/2	0.2 (0.1–0.4)	4.8 (2.2–9.2)	34/3	0.6 (0.4–0.8)	10.6 (7.3–14.8)
Lung, bronchi, and trachea	23/4	1.2 (0.8–1.7)	6.2 (3.9–9.3)	62/8	1.6 (1.3–2.1)	7.9 (6.0–10.1)	72/13	1.2 (1.0–1.5)	5.5 (4.3–6.9)
Ovary	–	–	–	8/1	0.4 (0.2–0.8)	9.5 (4.1–18.6)	10/1	0.3 (0.2–0.6)	7.2 (3.5–13.3)
Kidney	6/1	0.3 (0.1–0.6)	11.0 (4.0–24.0)	6/1	0.2 (0.1–0.3)	5.3 (2.0–11.6)	8/2	0.1 (0.1–0.3)	4.4 (1.9–8.7)
Non-Hodgkin lymphoma	10/1	0.5 (0.3–0.9)	8.5 (4.1–15.7)	13/3	0.3 (0.2–0.6)	5.1 (2.7–8.7)	17/5	0.3 (0.2–0.5)	3.7 (2.2–6.0)
Metastases and nonspecified cancer in lymph nodes	9/1	0.5 (0.2–0.8)	12.8 (5.9–24.3)	14/2	0.4 (0.2–0.6)	8.8 (4.8–14.8)	32/3	0.5 (0.4–0.8)	11.8 (8.1–16.7)

Abbreviations: AR, absolute risk; CI, confidence interval; O/E, observed/expected; SIR, standardized incidence ratio.

a Numbers <5 are omitted to comply with data protection guidelines.

**Table 5:** Standardized incidence ratios for cancer in users of low-dose aspirin with venous thromboembolism during the second and subsequent years of follow-up

	New users		Short-term users		Long-term users	
Characteristics	O/E	SIR (95% CI)	O/E	SIR (95% CI)	O/E	SIR (95% CI)
All	286/232	1.2 (1.1–1.4)	459/400	1.1 (1.1–1.3)	543/491	1.1 (1.0–1.2)
Type of VTE						
Deep vein thrombosis	159/141	1.1 (1.0–1.3)	313/251	1.2 (1.1–1.4)	324/282	1.1 (1.0–1.3)
Pulmonary embolism	127/91	1.4 (1.2–1.7)	146/149	1.0 (0.8–1.2)	219/208	1.1 (0.9–1.2)
Sex						
Female	120/95	1.3 (1.1–1.5)	211/185	1.1 (1.0–1.3)	277/233	1.2 (1.1–1.3)
Male	166/137	1.2 (1.0–1.4)	248/215	1.2 (1.0–1.3)	266/258	1.0 (0.9–1.2)
Age at VTE diagnosis						
0–60 years	51/45	1.1 (0.8–1.5)	52/40	1.3 (1.0–1.7)	24/25	1.1 (0.6–1.5)
≥61 years	235/187	1.3 (1.1–1.4)	407/360	1.1 (1.0–1.3)	519/466	1.1 (1.0–1.2)
Year of VTE diagnosis						
2001–2006	165/130	1.3 (1.1–1.5)	220/180	1.2 (1.1–1.4)	162/136	1.2 (1.0–1.4)
2007–2012	103/85	1.2 (1.0–1.5)	187/174	1.1 (0.9–1.2)	270/240	1.1 (1.0–1.3)
2013–2017	18/17	1.0 (0.6–1.6)	52/46	1.1 (0.9–1.5)	111/115	1.0 (0.8–1.2)
Comorbidity burden a						
Low	128/109	1.2 (1.0–1.4)	150/136	1.1 (0.9–1.3)	130/122	1.1 (0.9–1.3)
Medium	132/101	1.3 (1.1–1.6)	245/205	1.2 (1.1–1.4)	287/254	1.1 (1.0–1.3)
High	26/22	1.2 (0.8–1.7)	64/58	1.1 (0.9–1.4)	126/115	1.1 (0.9–1.3)
Provoking factor present b						
No	208/167	1.2 (1.1–1.4)	354/317	1.1 (1.0–1.2)	406/380	1.1 (1.0–1.2)
Yes	78/65	1.2 (1.0–1.5)	105/83	1.3 (1.0–1.5)	137/111	1.2 (1.0–1.5)
Cancer stage						
Localized	157/117	1.3 (1.1–1.6)	212/201	1.1 (0.9–1.2)	287/248	1.2 (1.0–1.3)
Nonlocalized	52/55	0.9 (0.7–1.2)	106/93	1.1 (0.9–1.4)	107/109	1.0 (0.8–1.2)
Unknown	77/60	1.3 (1.0–1.6)	141/106	1.3 (1.1–1.6)	149/134	1.1 (0.9–1.3)

Abbreviations: CI, confidence interval; O/E, observed/expected; SIR, standardized incidence ratio; VTE, venous thromboembolism.

a Based on Charlson's comorbidity index scores (low: 0, medium: 1–2, and high ≥3).

b Selected provoking factors were pregnancy, trauma/fractures, and surgery.

#### Short-Term Users

Among short-term users of low-dose aspirin, we observed 715 cancers, including 256 during the first year after VTE diagnosis. The corresponding 1-year absolute risk of cancer was 6.7% ( Table 2 ). The absolute risk was even higher for patients >60 years old at VTE diagnosis and patients with low comorbidity burden.

The SIR for short-term aspirin users was 3.2 (95% CI: 2.9–3.7) during the first year of follow-up ( Table 3 ). The highest SIR was observed for patients with low comorbidity burden. The site-specific SIRs during the first year of follow-up were highest for cancers of the liver, lung, ovary, and kidney, as well as the non-Hodgkin lymphoma ( Table 4 ). The overall SIR decreased to 1.1 (95% CI: 1.1–1.3) in the subsequent years of follow-up ( Table 5 ).

#### Long-Term Users

Among long-term users of low-dose aspirin, we observed 937 cancers, including 394 during the first year of follow-up, yielding an absolute 1-year risk of 6.7% ( Table 2 ). The absolute risk was even higher for patients >60 years old at VTE diagnosis, patients with low comorbidity burden, and patients without the selected provoking factors.

The SIR for long-term aspirin users was 2.8 (95% CI: 2.6–3.2) during the first year of follow-up and generally highest for patients with low comorbidity burden and patients without the selected provoking factors ( Table 3 ). The site-specific SIRs during the first year of follow-up were highest for cancers of the pancreas, lung, ovary, and kidney ( Table 4 ). The overall SIR decreased to 1.1 (95% CI: 1.0–1.2) in the subsequent years of follow-up ( Table 5 ).

The SIRs for nonlocalized cancer were markedly higher than for localized cancer in all three cohorts of aspirin users during the first year of follow-up ( Table 3 ). Both DVT and PE were clearly associated with increased cancer risk in all cohorts of aspirin users during the first year of follow-up ( Table 3 ). SIRs for both DVT and PE decreased to slightly above 1.0 in the subsequent years of follow-up ( Table 5 ).

#### Gastrointestinal Cancers

The majority of gastrointestinal cancers diagnosed during the first year of follow-up in all three cohorts of aspirin users were colorectal cancers (new users, SIR: 4.8 [95% CI: 2.5–8.2]; short-term users, SIR: 4.0 [95% CI: 2.6–6.0]; and long-term users, SIR: 3.5 [95% CI: 2.5–4.8]; Table 4 ). For cancers of the rectum, the SIRs were 2.5 (95% CI: 1.0–5.1) for short-term users and 2.1 (95% CI: 1.0–3.8) for long-term users. For cancers of the esophagus and stomach, the SIRs were 3.5 (95% CI: 1.1–8.1) and 2.7 (95% CI: 0.9–6.3), respectively, for long-term users. Due to the small numbers of cancers, we were not able to examine all cancer sites of the gastrointestinal tract for each of the cohorts.

### Number Needed to Examine

In the cohort of new users of low-dose aspirin, 2,003 patients with VTE would have to be examined to detect 85 excess cases of cancer during the first year after VTE. For short-term users, 3,851 patients with VTE would have to be examined to detect 177 excess cases of cancer. For long-term users, 5,905 patients would have to be examined to detect 256 excess cases of cancer. When the difference between the observed number of cancers and expected number of cancers was divided by the follow-up time, the corresponding number of patients who needed to be examined to detect one excess case of cancer was 20 (95% CI: 18–22) for new users, 17 (95% CI: 16–19) for short-term users, and 18 (95% CI: 16–20) for long-term users.

The number of patients with VTE needed to examine to detect one excess case of localized cancer was higher than the number of patients with VTE needed to examine to detect one excess case of nonlocalized cancer in all three cohorts of aspirin users (238 for localized cancer vs. 49 for nonlocalized cancer among new users, 170 vs. 34 among short-term users, and 124 vs. 35 among long-term users).

## Discussion

In this population-based cohort study of aspirin users, patients with a diagnosis of VTE had an approximately 6% absolute risk of a cancer diagnosis during the first year of follow-up. This corresponded to a three-fold increased risk of cancer compared with the general population. The increased cancer risk was observed in new, short-term, and long-term users of low-dose aspirin. In all three cohorts of aspirin users, we observed an increased risk of gastrointestinal cancers, particularly colorectal cancers. The excess risk of cancer decreased in subsequent years but remained slightly elevated. The number of VTE patients who needed to be examined to detect one excess case of cancer within the first year after VTE diagnosis was 17 to 20, assuming that cancers were present at the time of VTE diagnosis.

Our results suggest that aspirin users with a diagnosis of VTE have an increased risk of a subsequent cancer diagnosis, similar to or slightly higher than that observed in previous studies investigating patients with VTE in the general population 5 6 which may reflect the fact that occult cancer promotes VTE in aspirin users. The increased risk varied according to cancer site which is broadly similar to observations in previous studies. Our results are also consistent with the increased cancer risk after a VTE event among patients with acute myocardial infarction or stroke. 17 The reasons for the slightly elevated cancer risk beyond the first year of follow-up are not clear, but physiological factors associated with thrombosis, such as prostaglandins, have been suggested to promote cancer. 4 Furthermore, established shared lifestyle risk factors, such as use of oral contraceptives, smoking, and obesity, may partially reflect the long-term elevated risk. 1 4

Our study aimed to examine whether a diagnosis of VTE remains a marker of incident of cancer in aspirin users. Aspirin use has been found to reduce the risk of cancer, particularly gastrointestinal cancer, 10 11 and has been recommended for the primary prevention of colorectal cancer. 34 However, our findings suggest that aspirin use does not appear to affect the increased risk of cancer, including gastrointestinal cancers, after a diagnosis of VTE. The cancer-protective effects of aspirin may be attributable to the induction of cellular apoptosis and inhibition of cyclooxygenase, 13 but aspirin may also have indirect effects. Thus, a recent study showed that initiation of aspirin use may reduce colorectal cancer risk by increasing the risk of bleeding from premalignant colorectal polyps, leading to colonoscopy and polypectomy before the manifestation of colorectal cancer. 35

## Strengths and Limitations

The strengths of our study include its nationwide population-based design with access to virtually complete follow-up of all patients, reducing the risk of referral bias. The validity of a first-time VTE diagnosis in the DNPR is high, with a positive predictive value of 90%. 36 Moreover, the Danish registry data on cancer diagnoses, 21 prescriptions, 20 surgery codes, 19 and comorbidities 27 are of high quality.

A potential limitation of our study is the lack of information on over-the-counter use of aspirin. However, approximately 90% of low-dose aspirin sales in Denmark are prescribed. 23 Therefore, any misclassification due to over-the-counter use of aspirin probably did not affect our results. Another limitation is the use of prescription data to estimate low-dose aspirin use which may have resulted in misclassification of short-term aspirin use due to nonadherence. However, in Denmark, the correspondence to dispensation within±90 days of general practitioner-reported use of low-dose aspirin is as high as 93%. 37

The likelihood of detecting cancers in VTE patients may be increased during a hospital contact due to increased diagnostic surveillance. The markedly increased risk in the first year after diagnosis of VTE and the diminishment of excess risk thereafter is consistent with this explanation. However, detection bias is unlikely to fully explain our results; the cancer risk beyond 1 year after VTE diagnosis remained slightly elevated, and we observed no compensatory deficit 6 (i.e., we observed no decreased risk of cancer beyond 1 year of follow-up after the initial period of increased cancer risk in the first year after VTE diagnosis).

Cancers diagnosed within 1 year after a VTE event are associated with an advanced stage of cancer and poor prognosis. 2 This is in accordance with our findings which suggest a clearly higher SIR for nonlocalized cancers than for localized cancers. These findings also argue against detection bias, as we would have expected the diagnosis of more localized cancers rather than nonlocalized cancers if patients with VTE had heightened surveillance for cancer. In addition, we found that up to 238 patients with VTE would have to be examined in order detect one excess case of localized cancer which was markedly higher than for nonlocalized cancers. This may indicate that more nonlocalized cancers than localized cancers would be detected if extensive diagnostic workups for cancer were initiated within the first year after a VTE event, making it unclear whether an extensive workup after a VTE event would improve patient outcomes.

## Conclusion

In our study, 17 to 20 patients with VTE would have to be examined to detect one excess case of cancer during the first year of follow-up. However, the effectiveness of extensive cancer screening depends on the ability of the screening to detect a greater number of cancers and improve patient prognosis due to the early detection 38 which we did not investigate. Thus, the clinical implications for extensive cancer screening are unclear. Only a few randomized trials have compared extensive and limited screening for cancer among patients with primary VTE. 39 40 41 In these studies, extensive cancer screening (e.g., computed tomography of the chest, abdomen, and pelvis) was not associated with improved patient prognosis. In addition, extensive cancer screening may be harmful and cause unnecessary patient anxiety. 42 These findings are in accordance with the latest National Institute for Health and Care Excellence guidelines 43 which do not recommend extensive screening for cancer in patients with unprovoked VTE unless these patients have relevant clinical symptoms or signs. These same guidelines could be applied to users of low-dose aspirin with VTE.

In conclusion, VTE may be a marker of occult cancer, even in users of low-dose aspirin, regardless of the duration of use.

## Notes

What Is Known on This Topic?

- Venous thromboembolism can be the first symptom of occult cancer.
- Aspirin may reduce the risk of cancer, particularly gastrointestinal cancer, and venous thromboembolism.
- Whether venous thromboembolism is a marker of occult cancer in aspirin users is unknown.

**What Does This Paper Add?**

- Venous thromboembolism may be a marker of occult cancer, including gastrointestinal cancer, in users of low-dose aspirin, regardless of duration of use.
- The increased risk of cancer in the first year after venous thromboembolism was higher for nonlocalized cancers than localized cancers.
- Guidelines for cancer screening in patients with unprovoked venous thromboembolism could be applied to users of low-dose aspirin with venous thromboembolism.
