# Supplementary material for: Venous Thromboembolism and Risk of Cancer in Users of Low-Dose Aspirin: A Danish Population-Based Cohort Study
Source: TH Open. 2022 Sep 12;6(3):e257–66. doi: 10.1055/s-0042-1755606 (PMC9467693; doi:10.1055/s-0042-1755606)
Supplement: Supplementary file 1 — Supplementary Material [file 10-1055-s-0042-1755606-s220026.pdf]

Supplementary Table S1 Data sources

The Danish National Patient Registry contains information on all nonpsychiatric hospitalizations since 1977 and all outpatient hospital and emergency room contacts since 1995.[19] Each record contains data on the civil registration number, dates of admission and discharge, codes for surgeries, procedures, and treatments, and 1 primary diagnosis and up to 20 secondary diagnoses coded according to the International Classification of Diseases (ICD) 8th revision until the end of 1993 and the 10th revision thereafter. Surgical procedures have been coded according to the Nordic Medico-Statistical Committee (NOMESCO) Classification of Surgical Procedures since 1996.
The Danish National Prescription Registry includes data on all prescriptions redeemed at Danish community pharmacies since 1995. Data in the prescription registry include the patient's civil registration number and the dosage, type, and quantity of all prescription medications. The medications are coded according to the Anatomical Therapeutic Chemical Classification System.[20]
The Danish Cancer Registry contains data on all incident malignant neoplasms diagnosed in Denmark since 1943. Data include civil registration number, date of cancer diagnosis, and type, location, and stage at the time of diagnosis. Since 1978, tumor characteristics in the Danish Cancer Registry have been coded according to the 10th revision of the ICD.[21]

Supplementary Table S2 Codes used in the Danish National Patient Registry, Danish Cancer Registry, and Danish National Prescription Registry

	ICD-8[a]	ICD-10[a]	ATC codes
Venous thromboembolism			
Deep vein thrombosis	451.00	I801, I802, I803	
Pulmonary embolism	450.99	I26	
Select provoking factors			
Pregnancy		O00-O99	
Trauma/fractures		S00-T14	
Surgical procedures (excluding minor procedures)		Procedure codes (NOMESCO): All K codes except KA-KQ, KX, KY	
Selected comorbidities			
Ischemic stroke	433; 434	I63; I64	
Myocardial infarction	410	I21	
Diabetes mellitus (type 1 and type 2)	249; 250	E10; E11	A10A; A10B
Cancers		ICD-10	
All cancer diagnoses		C00–C96	
Lung, bronchi, and trachea		C33–C34	
Colon, incl. colon rectosigmoid		C18–C19	
Pancreas		C25	
Basal cell carcinoma		C44	
Prostrate		C61	
Non-Hodgkin lymphoma		C82–C90	
Non-specified cancer in lymph nodes		C77–C79	
Urinary bladder		C67	
Breast		C50	
Ovary		C56, C570–C574	
Kidney		C64	
Other skin cancers		C44	
Esophagus		C15	
Stomach		C16	
Rectum		C20–C21	

(*Continued*)

 © 2022. The Author(s).

Supplementary Table S2 (*Continued*)

	ICD-8[a]	ICD-10[a]	ATC codes
Liver		C22	
Gallbladder and biliary tract		C23–C24	
Uterus		C54–C55	
Non-specified cancers		C76, C80	
Lymphoid leukemia		C91	
Myeloid leukemia		C92	
Malignant melanoma		C43, C21 (morphology 872–879)	
Prescriptions			
Aspirin codes			B01AC06 (75 mg) + N02BA01 (100, 150 mg)

Abbreviations: ATC, Anatomical Therapeutic Chemical; ICD, International Classification of Diseases; NOMESCO, Nordic Medico-Statistical Committee.
[a]The listed ICD codes contain all subgroups of codes.

Supplementary Table S3 Codes used for cancer stage in the Danish Cancer Registry (DCR)

	DCR codes before 2004	DCR codes from 2004 onwards			
	Codes	TNM stage	Codes-T	Codes-N	Codes-M
Localized	0, 1, 2, 5	T1–4, x N0 M0	AZCD13–19	AZCD30	AZCD40
		T1–2 N0 Mx	AZCD13–14	AZCD30	AZCD49
		T1 Nx M0,x	AZCD13	AZCD39	AZCD40, 49
Nonlocalized	3, 4, 6, 7	T1–4, x N1–3 M0–1, x	AZCD13–19	AZCD31–33	AZCD40, 41, 49
		T1–4, x N0 M1	AZCD13–19	AZCD30	AZCD41
		T1–4, x Nx M1	AZCD13–19	AZCD39	AZCD41
Unknown	A, B, 9	T2–4, x Nx M0, x	AZCD14–19	AZCD39	AZCD40, 49
		T3–4, x N0 Mx	AZCD15–19	AZCD30	AZCD49
		T0, a, is, N0–3, x M0–1, x	AZCD10–12	AZCD30–33, 39	AZCD40–41, 49

Abbreviation: TNM, tumor, nodes, and metastases according to Union for International Cancer Control TNM classification system of malignant tumors.

 © 2022. The Author(s).

Supplementary Table S4 Modified Charlson's comorbidity index excluding any previous tumors before the venous thromboembolism diagnosis

	Diseases	ICD-8	ICD-10	Score
1	Myocardial infarction	410	I21;I22;I23	1
2	Congestive heart failure	427.09; 427.10; 427.11; 427.19; 428.99; 782.49	I50; I11.0; I13.0; I13.2	1
3	Peripheral vascular disease	440; 441; 442; 443; 444; 445	I70; I71; I72; I73; I74; I77	1
4	Cerebrovascular disease	430–438	I60-I69; G45; G46	1
5	Dementia	290.09–290.19; 293.09	F00-F03; F05.1; G30	1
6	Chronic pulmonary disease	490–493; 515–518	J40-J47; J60-J67; J68.4; J70.1; J70.3; J84.1; J92.0; J96.1; J98.2; J98.3	1
7	Connective tissue disease	712; 716; 734; 446; 135.99	M05; M06; M08; M09; M30; M31; M32; M33; M34; M35; M36; D86	1
8	Ulcer disease	530.91; 530.98; 531–534	K22.1; K25-K28	1
9	Mild liver disease	571; 573.01; 573.04	B18; K70.0-K70.3; K70.9; K71; K73; K74; K76.0; DB18	1
10	Diabetes type 1	249.00; 249.06; 249.07; 249.09	E10.0, E10.1; E10.9	1
	Diabetes type 2	250.00; 250.06; 250.07; 250.09	E11.0; E11.1; E11.9	
11	Hemiplegia	344	G81; G82	2
12	Moderate-to-severe renal disease	403; 404; 580–583; 584; 590.09; 593.19; 753.10–753.19; 792	I12; I13; N00-N05; N07; N11; N14; N17-N19; Q61	2
13	Diabetes with end-organ damage			2
	Type 1	249.01–249.05; 249.08	E10.2-E10.8	
	Type 2	250.01–250.05; 250.08	E11.2-E11.8	
14	Moderate-to-severe liver disease	070.00; 070.02; 070.04; 070.06; 070.08; 573.00; 456.00–456.09	B15.0; B16.0; B16.2; B19.0; K70.4; K72; K76.6; I85	3
15	AIDS	079.83	B21-B24	6
	Excluded from the original definition			
16	Any previous tumors	140–194	C00-C75	2
17	Leukemia	204–207	C91-C95	2
18	Lymphoma	200–203; 275.59	C81-C85; C88; C90; C96	2
19	Metastatic solid tumor	195–198; 199	C76-C80	6

Abbreviation: ICD, International Classification of Diseases.

 © 2022. The Author(s).
